# Placental, hepatic, and supraclavicular lymph node metastasis in pancreatic adenocarcinoma during pregnancy: A case report

**DOI:** 10.4274/tjod.39327

**Published:** 2016-09-15

**Authors:** Seda Şahin Aker, Doruk Cevdi Katlan, Tuncay Yüce, Feride Söylemez

**Affiliations:** 1 Dr. Sami Ulus Maternity and Children’s Health Training and Research Hospital, Clinic of Gynecology and Obstetrics, Ankara, Turkey; 2 Ankara University Faculty of Medicine, Department of Obstetrics and Gynecology, Ankara, Turkey

**Keywords:** Pancreatic carcinoma, malignancy, Pregnancy

## Abstract

The occurrence of coexisting cancer in pregnant women is not a common phenomenon. It complicates approximately 1 in 1000 to 1500 pregnancies. We present a multiparous woman aged 27 years in her 28^th^ week of pregnancy who was admitted to our clinic with right upper quadrant pain and was finally revealed to have multiple metastatic pancreatic adenocarcinoma. To the best of our knowledge, this is the first documented case of pancreatic adenocarcinoma to metastasize both to the placenta and multiple maternal sites (liver, supraclavicular, para-aortic lymph nodes) in a pregnant patient. Unpredictable metastases to the placenta may be encountered and may even lead to definitive diagnosis, as in our case. Therefore, the placenta in any patient with known malignancy should be sent for pathologic evaluation.

## INTRODUCTION

The occurrence of coexisting cancer in pregnant women is not a common phenomenon. It complicates approximately 1 in 1.000 to 1.500 pregnancies^(1)^. It is estimated that about 3.500 new cases of cancer are diagnosed annually in pregnant women in the United States of America, which corresponds to 1 case for every 1.000 gestations^(2)^. The most frequently diagnosed malignancies in pregnancy are breast cancer, cervical cancer, malignant melanoma, and lymphomas, respectively^(3)^. The most common tumor that metastasizes to the fetus or placenta is malignant melanoma, which accounts for 30% of all pregnancy-associated tumors^(4)^. The second most frequently metastasizing malignancies are leukemia and lymphoma, followed by carcinoma of the breast and lung^(4)^. Herein, we present a multiparous woman aged 27 years in her 28^th^ week of pregnancy who was admitted to our clinic with right upper quadrant pain and was finally revealed to have multiple metastatic pancreatic adenocarcinoma.

## CASE REPORT

A multiparous patient aged 27 years in her 26^th^ week of gestation presented with right upper quadrant pain, nausea, and vomiting. The patient was admitted to the hospital with a preliminary diagnosis of acute cholecystitis. Murphy’s sign was positive. There were no signs of acute peritoneal irritation and she was initially managed with a nil-by-mouth status, intravenous hydration, and antispasmodic medication for abdominal cramps. She had a serum indirect bilirubin titre of 0.7 mg/dL (direct 0.2 mg/dL), and alkaline phosphatase titre of 123 u/L. Serology against viral hepatitis antigens were all negative. Levels of cancer antigen (Ca) 19-9, Ca 125, and alpha-fetoprotein tumor markers were all elevated to >2.064 U/mL, 985 U/mL, and 45 ng/mL respectively. Obstetric ultrasonography revealed an intrauterine male fetus with normal biometric measurements and biophysical profile scores. Right upper-quadrant abdominal ultrasound revealed a normal gallbladder, no dilatation of the common bile duct, and no periportal mesenteric pathologic lymphadenopathy. However, cervical ultrasound revealed bilateral cervical and left supraclavicular conglomerate lymphadenopathy. Supracervical lymph node excisional biopsy was performed. The pathologic diagnosis was reported to be anaplastic carcinoma, possibly originating from the gastrointestinal tract, gallbladder or pancreas. Due to relatively advanced gestational age of the fetus and poor prognosis of the mother if left untreated, corticosteroids were administered to enhance fetal lung maturity and cesarean section was performed at 28 weeks to deliver a 1.000-g male fetus with Apgar scores of 6-8, 1-5 minutes. The neonate was transferred to the neonatal intensive care unit but died of sepsis 30 days post-partum. The family refused autopsy of the neonate and mother; therefore, no pathologic condition could be observed in the neonate.

**Figure 1:**
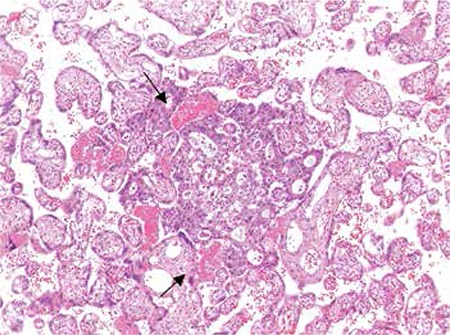
Small cribriform-forming atypical tumoral cells in placental chorionic villi

**Figure 2:**
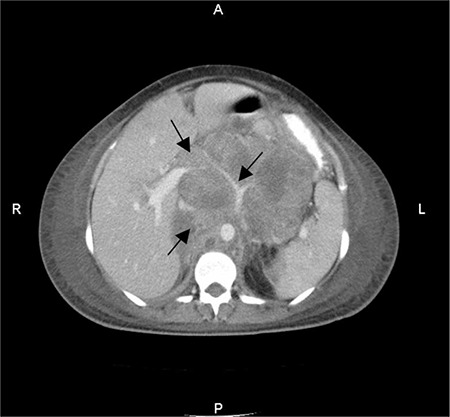
Axial contrast-enhanced computed tomographic image reveals a 13x11 cm mass located between the liver and para-aortic area

Intraperitoneal massive ascites was observed perioperatively, a sample of which was sent to pathologic examination together with the placenta. Pathology revealed malignancy-positive ascites cytology and metastasis of pancreatic adenocarcinoma to the placenta (Figure 1). Postoperatively, the patient was referred to medical oncology. Abdominopelvic computerized tomography (CT) demonstrated cervical, mediastinal, hilar, mesenteric, inguinal, parailiac and para-aortic multiple lymphadenopathies. The pancreatic head was impossible to visualize because of massive lymphadenopathy and a metastatic 6x7 cm mass was observed in the caudal lobe of liver (Figure 2). The patient experienced cardiac arrest during the immediate post CT period but returned to sinus rhythm after proper resuscitation. However, she succumbed to her cancer after two weeks. In this case, no familial cancer syndromes, cigarette smoking, genetic tendency or diabetes mellitus were identified, and there were no known toxic exposure.

## DISCUSSION

Cancer-complicated pregnancy is a rare coexistence and presents in approximately 1 in 1.000 to 1.500 pregnancies. Among those, pancreatic cancer is even rarer in a pregnant patient^(5)^. Risk factors for pancreatic cancer include cigarette smoking, advanced age, male sex, African-American ethnicity, family history, genetic background/tendency, diabetes mellitus, and chronic pancreatitis^(5)^. The most common presenting symptoms in patients with pancreatic cancer are abdominal and epigastric pain, dark urine, jaundice, nausea, back pain, diarrhoea, and vomiting^(6)^. Ultrasound and magnetic resonance imaging are the mainstays of imaging during pregnancy. Other options may be used if necessary. Fetal outcomes are generally very good, with iatrogenic prematurity being the most common complication. Surgical resection followed by adjuvant therapy is the standard of care for early-stage disease. Surgical intervention in the first trimester carries a risk of spontaneous abortion, and the size of the uterus is prohibitive in the third trimester; therefore, the second trimester is the ideal time for tumor resection^(7)^. Only 24 cases of pancreatic adenocarcinoma diagnosed antepartum have been described in the literature. The gestational age of the patients range from 4 to 36 weeks^(8)^. Khatsiev et al.^(9)^ reported a case of pancreatic adenocarcinoma in a pregnant woman aged 29 years with situs inversus in the first trimester. Patient underwent pancreatoduodenectomy following abortion. Onuma et al.^(10)^ described a woman at 34 weeks gestation who presented with uterine contractions and a large retroperitoneal mass. Pancreatic cancer was detected after emergency cesarean delivery, and pancreatoduodenectomy was performed. Kakoza et al.^(7)^ described a woman who was diagnosed as having cholecystitis and gallstone pancreatitis at 24 weeks gestation. Our patient presented as having acute cholecystitis.

Vertical transmission of cancer is exceptionally rare, although maternal cells do reach the fetus. From 1866 to 1999, 58 cases of documented maternal malignancy metastatic to the placenta and fetus were reported in the English literature^(11)^. The most common tumor that metastasizes to the fetus or placenta is malignant melanoma, which accounts for 30% of all pregnancy-associated tumors. The second most frequently metastasizing malignancies are leukemia and lymphoma, followed by carcinoma of the breast and lung^(12)^. The liver and peritoneal cavities are the most common sites of metastases in pancreatic cancer. Marci et al reported a case of pancreatic carcinoma in a women at 35 weeks gestation with multiple liver metastasis^(13)^. Metastases to the placenta and supraclavicular lymph nodes are extremely unusual. A review of the literature revealed only six cases of supraclavicular metastases of pancreatic adenocarcinoma^(14)^. Al-Adnani et al.^(15)^ reported a case of maternal pancreatic carcinoma metastatic to the placenta. In our case, placental and multiple maternal metastases were documented. Metastatic disease to the fetus is incredibly rare and has been documented only in melanoma, leukemia, and lymphoma.

To the best of our knowledge, this is the first documented case of a pancreatic adenocarcinoma to metastasize both to the placenta and multiple maternal sites (liver, supraclavicular, para-aortic lymph nodes) in a pregnant patient. Unpredictable metastases to the placenta may be encountered and may even lead to definitive diagnosis, as in our case. Therefore, the placenta in any patient with known malignancy should be sent for pathologic evaluation. Moreover, optimal care of a pregnant woman with metastatic cancer requires a prompt multidisciplinary approach, with special focus on maternal wellbeing and survival, while trying to minimize the negative impact on the fetus secondarily.
